# Synergistic effect of BCL2 and FLT3 co-inhibition in acute myeloid leukemia

**DOI:** 10.1186/s13045-020-00973-4

**Published:** 2020-10-19

**Authors:** Lindsey T. Brinton, Pu Zhang, Katie Williams, Daniel Canfield, Shelley Orwick, Steven Sher, Ronni Wasmuth, Larry Beaver, Casey Cempre, Jordan Skinner, Matthew Cannon, Mukul Govande, Bonnie Harrington, Amy Lehman, John C. Byrd, Rosa Lapalombella, James S. Blachly

**Affiliations:** 1 Division of Hematology, Department of Internal Medicine, The Ohio State University, Columbus, OH USA; 2 Department of Biomedical Engineering, The Ohio State University, Room 455C, OSUCCC Building, 410 West 12th Avenue, Columbus, OH 43210 USA; 3 College of Veterinary Medicine, Michigan State University, Lansing, MI USA; 4 Center Biostatistics, The Ohio State University, Columbus, OH USA; 5 College of Pharmacy, The Ohio State University, Columbus, OH USA; 6 College of Veterinary Medicine, The Ohio State University, Columbus, OH USA; 7 Leukemia Research Program, The Ohio State University James Comprehensive Cancer Center, Columbus, OH USA; 8 Department of Biomedical Informatics, The Ohio State University, Columbus, OH USA

**Keywords:** Gilteritinib, Midostaurin, Synergy, Combination therapy, FLT3, BCL2, Venetoclax, Acute myeloid leukemia

## Abstract

Acute myeloid leukemia (AML) is a heterogeneous and complex disease, and treatments for this disease have not been curative for the majority of patients. In younger patients, internal tandem duplication of *FLT3* (*FLT3*-ITD) is a common mutation for which two inhibitors (midostaurin and gilteritinib) with varied potency and specificity for FLT3 are clinically approved. However, the high rate of relapse or failed initial response of AML patients suggests that the addition of a second targeted therapy may be necessary to improve efficacy. Using an unbiased large-scale CRISPR screen, we genetically identified BCL2 knockout as having synergistic effects with an approved FLT3 inhibitor. Here, we provide supportive studies that validate the therapeutic potential of the combination of FLT3 inhibitors with venetoclax in vitro and in vivo against multiple models of *FLT3*-ITD-driven AML. Our unbiased approach provides genetic validation for co-targeting FLT3 and BCL2 and repurposes CRISPR screening data, utilizing the genome-wide scope toward mechanistic understanding.

Acute myeloid leukemia (AML) is a molecularly complex disease due to the presence of multiple genetic abnormalities that influence prognosis and therapy outcome [1]. Several approaches for improving outcomes in AML patients have been investigated to date with moderate success. Internal tandem duplications (ITDs) in the *FMS-like tyrosine kinase 3* (*FLT3*) gene are among the most common abnormalities seen in younger adult AML and are present in approximately 30% of patients; it leads to constitutive FLT3 kinase activity and downstream activation of signal transducer and activator of transcription 5 (STAT5) [2, 3]. The presence of *FLT3*-ITD mutations at a high ITD/wild-type ratio has prognostic value both as a risk factor for relapse and poor survival in AML patients [4]. FLT3-targeting inhibitors that block constitutively active FLT3 kinase and downstream proliferative signaling have been developed and tested clinically; these include midostaurin and gilteritinib [5–9]. Despite the initial success at prolonging survival rates compared to prior standards therapies, available follow-up data with FLT3 inhibitor therapies show that this class of drug is plagued by short duration of response and nearly inevitable relapse when given combined with other AML therapies [7, 10]. This suggests that alternative combination approaches are required to fully appreciate optimal disease control in AML.

We have recently published an unbiased large-scale CRISPR (clustered regularly interspaced short palindromic repeats) screen in which we focused on a predicted novel combination strategy of the FLT3 inhibitor midostaurin with the XPO1 (Exportin 1) inhibitor selinexor; however, our data also predicted synergy between midostaurin and genetic *B-cell lymphoma *2 (*BCL2*) knockout [11] (Fig. 1a), prompting further validation study of the combination of FLT3 and BCL2 inhibitors. Previous work by Ma et al. [12] also showed the in vitro effects of combining FLT3 and BCL2 inhibitors. This work stemmed from the observation that FLT3 inhibitors result in downmodulation of the expression of myeloid cell leukemia 1 (MCL1), a known mechanism of resistance to BCL2 inhibitors, and could improve activity of BCL2 inhibitors. Mali et al. also conducted a series of studies using the second-generation FLT3 inhibitor quizartinib [which additionally inhibits KIT and platelet-derived growth factor receptors (PDGFR)] [13] in combination with venetoclax [14]. Unlike gilteritinib, quizartinib is a type II inhibitor, interacting only with the inactive conformation and therefore is not effective against a FLT3-tyrosine kinase domain (TKD) mutation; quizartinib was denied FDA approval in June 2019. Because the inhibitors interact with different conformations, resistance patterns differ between patients treated with type 1 inhibitors like gilteritinib and type II like quizartinib, with the later often acquiring a resistance-causing FLT3-TKD mutation during therapy [15]. However, in vivo synergism studies by Mali et al. support our combination strategy and their mechanistic studies informed our pathway analysis design. Complementary to studies by these groups [12, 14], our unbiased approach provides genetic validation for targeting these genes, assures that the mechanisms underlying the synergy are sound, and extends preclinical work advancing clinical translatability of this combination—including demonstrating the combination of FDA-approved FLT3 inhibitors with venetoclax in vivo against multiple models of *FLT3*-ITD-driven AML beyond the previously studied MV4-11 xenograft model. The high translatability of this combination therapy stands to make a rapid clinical impact for AML patients, especially considering the high relapse rate associated with FLT3 inhibitors. Our approach also illustrates that CRISPR screening is a noteworthy method for identifying rational combination strategies for genetic subsets of AML.

**Fig. 1:**
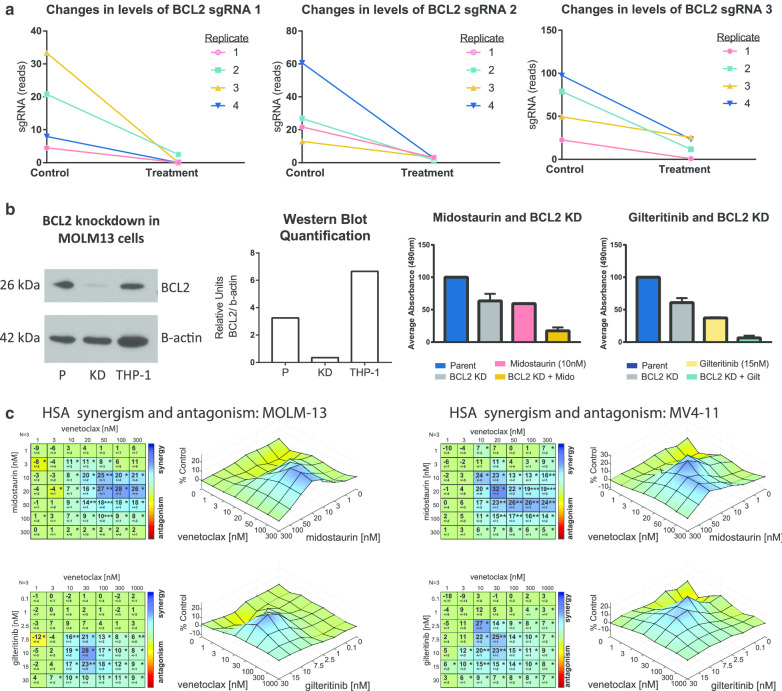
In vitro genetic and pharmacologic validation in *FLT3*-ITD cell lines. **a** Changes in reads of sgRNAs targeting BCL2 in a CRISPR knockout screen with midostaurin. Three guides are targeted to BCL2 in each of four replicate screens. **b** BCL2 expression was knocked down in MOLM-13 cells (BCL2 KD), confirmed via immunoblot using THP-1 as a positive control for BCL2 expression, and proliferation changes compared to parental cells (P) under treatment with 10 nM midostaurin, 15 nM gilteritinib, or control DMSO. A mixed effects model was applied to the data. Compared to DMSO, the average decrease in absorbance with midostaurin + BCL2 KD was larger than the observed decrease for midostaurin in parental cells (estimated difference = − 0.35; 95% CI − 0.49, − 0.2; *p* = 0.002) or with BCL2 KD alone (estimated difference: − 0.37; 95% CI − 0.4, − 0.33; *p* < .001). Similarly, the average decrease in absorbance for gilteritinib + BCL2 KD was larger than the decreases in absorbance for gilteritinib/parental (− 0.24; 95% CI − 0.31, − 0.17; *p* < .001) or BCL2 KD (− 0.42; 95% CI − 0.45, − 0.39; *p* < .001). **c** *FLT3*-ITD cell lines MOLM-13 and MV4-11 were treated with a range of doses of either midostaurin plus venetoclax or gilteritinib plus venetoclax for 48 h, and then, MTS reagent was added and absorption read. Highest single-agent (HSA) analysis was used to determine regions of synergy. *p < 5 × 10^–2^, **p < 10^–3^, ***p < 10^–4^

Results from our published CRISPR screen with midostaurin in the *FLT3*-ITD cell line MOLM-13 [11] revealed the apoptosis regulator *BCL2* among several other genes whose knockout improved the effect of midostaurin. Based on these results, we examined in vitro and in vivo relevance of the loss of BCL2 expression on FLT3 inhibitor-mediated cell death. We first performed knockdown (KD) of BCL2 expression with CRISPR ribonucleoprotein in MOLM-13 using two different guides and confirmed reduced protein expression via immunoblot analysis (Fig. 1b). Next, we tested whether BCL2 KD increased the effect of midostaurin and also an alternative gilteritinib-based treatment. As shown in Fig. 1b, midostaurin or BCL2 KD alone decreased viable cells, but the combination of FLT3 inhibition with BCL2 KD strongly decreased viability over midostaurin alone (*p* < 0.01) or BCL2 KD alone (*p* < 0.001). The combination of BCL2 KD with gilteritinib demonstrated a similar effect, where BCL2 knockdown and gilteritinib in combination significantly decreased viability compared to gilteritinib alone (*p* < 0.001) or BCL2 KD alone (*p* < 0.001, Fig. 1b).

We next showed that synergistic effects could also be achieved using the clinically approved inhibitor of BCL2, venetoclax. Ma et al. examined apoptosis in a short time course (4, 6, 8, or 24 h), while we examined all anti-proliferative effects following a longer duration of treatment (48 h). As shown in Fig. 1c, the addition of venetoclax to midostaurin or gilteritinib exhibited synergism in the *FLT3*-ITD cell lines MOLM-13 and MV4-11, while limited to absent synergism was observed in *FLT3* wild-type (WT) AML cell lines (Additional file 1: Fig. S1A). This assay allowed us to map the level of synergism observed at different dose combinations in order to visualize the patterns of synergy and predict the dose combinations that exhibit the highest decrease in proliferation. In MOLM-13 cells, the most effective combination range was 20–300 nM venetoclax (peak 100 nM) with 10–50 nM midostaurin (peak 20 nM) or 7.5–15 nM gilteritinib (peak 10 nM) with 10–100 nM venetoclax (peak 30 nM). In MV4-11 cells, dose ranges were comparable: 10–300 nM venetoclax (peak 20 nM) with 10–50 nM midostaurin (peak 20 nM) or 2.5–10 nM gilteritinib (peak 2.5 nM) with 3–30 nM venetoclax (peak 10 nM). These doses of drug correspond to pharmacologic levels of these agents attained in vivo among AML patients being treated with these drugs.

To further characterize the anti-proliferative effects of midostaurin or gilteritinib in combination with venetoclax, we mimicked disease conditions by examining primary samples in a stromal cell co-culture system under hypoxic conditions for 96 h. Samples with *FLT3*-ITD responded more robustly to the combination of inhibitors than to either alone (Fig. 2a). Very limited enhanced effect with the addition of venetoclax to midostaurin was observed in *FLT3*-WT primary samples, as expected; however, the *FLT3*-WT samples examined here demonstrated synergy with gilteritinib/venetoclax combination treatment (Fig. 2b). While Ma et al. also observed a small increase in apoptosis in *FLT3*-WT samples, the proliferation assays indicate stronger synergism, suggesting that in the case of *FLT3*-WT, the activity of the drug combination may include cytotoxic, cytostatic, or some other non-apoptotic mechanism.

**Fig. 2:**
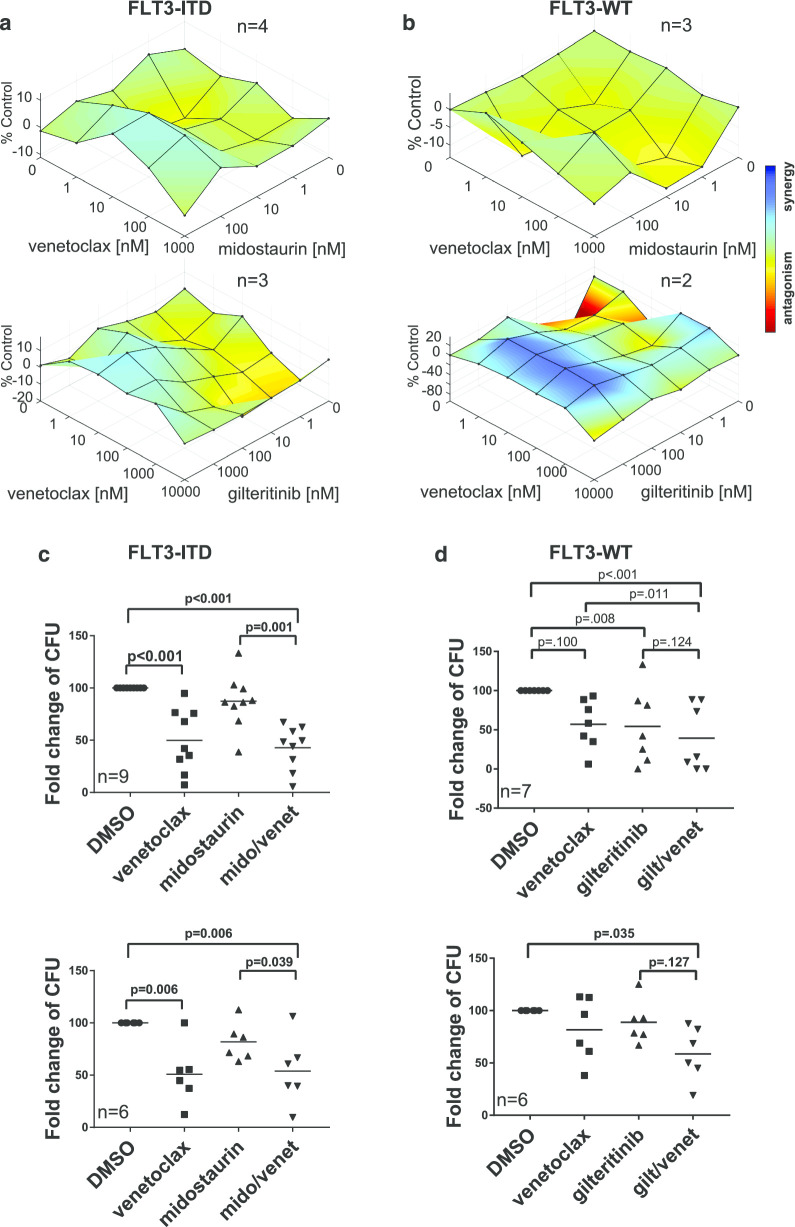
In vitro pharmacologic validation in primary patient samples. Primary samples from AML patients with **a** *FLT3*-ITD (*n* = 4 for midostaurin + venetoclax or *n* = 3 for gilteritinib + venetoclax) or **b** *FLT3*-WT (*n* = 3 for midostaurin + venetoclax or *n* = 2 for gilteritinib + venetoclax) were co-cultured with HS5 stromal cells and treated at a range of doses for 96 h. MTS reagent was added to blast cells, and absorption results averaged and analyzed by HSA. **c** *FLT3*-ITD or **d** *FLT3*-WT primary patient samples were cultivated in duplicate in Methocult media with control DMSO, 100 nM venetoclax, 100 nM midostaurin, 50 nM gilteritinib, combination of midostaurin and venetoclax, or combination of gilteritinib and venetoclax for 7–10 days, and then, colonies were counted. Results reported for midostaurin + venetoclax consist of 9 individual patients with *FLT3*-ITD AML and 6 individual patients with *FLT3*-WT AML and for midostaurin + venetoclax consist of 9 individual patients with *FLT3*-ITD AML and 6 individual patients with *FLT3*-WT AML

One way AML cells evade apoptosis is by up-regulation of pro-survival members of the BCL2 protein family, including BCL2, MCL1, and B-cell lymphoma-extra large (BCL-X_L_) [16]. These proteins bind BCL2-associated X protein (BAX) and BCL2 antagonist/killer (BAK), sequestering them to prevent apoptosis induction. Ma et al. demonstrate that FLT3 inhibitors downregulate MCL1, causing AML cells to rely more on the binding of BAX and BAK to BCL2 [12]. Therefore, when venetoclax is added to a FLT3 inhibitor, they suggest there is synergy because both MCL1 and BCL2 expressions are reduced, increasing the dampening of the pro-survival activity of the AML blasts. In their work with the combination of quizartinib and venetoclax, Mali et al. explored a similar mechanism in vivo in an MV4-11 xenograft model using inhibitors with different selectivities for each of the BCL2 family members in combination: venetoclax (BCL2), navitoclax (BCL2 and BCL-X_L_), and AMG 176 (MCL1) [14]. Their results indicated that the improvement in survival was highest when quizartinib was combined with a BCL2, and not MCL1, inhibitor. Furthermore, combination of quizartinib with venetoclax was superior to combination with navitoclax plus AMG 176, suggesting that quizartinib elicits anti-tumor activity outside of its reduction of MCL1 levels. In in vitro studies, they also did not see demonstrable synergy using A1331852 (BCL-X_L_ specific) with quizartinib [14]. Unlike our observations with gilteritinib, no synergism was observed in *FLT3*-WT using quizartinib, highlighting that there are likely differences in how these two drugs elicit their effects. We sought to further explore how different BCL2 family members act under FLT3 inhibition using our genome-wide CRISPR data.

Data from the midostaurin CRISPR screen show how single guide ribonucleic acid (sgRNA) levels change for each of the BCL2 family members, as well as for BAX and BAK while being treated with midostaurin (Additional file 1: Fig. S1B). In agreement with the mechanistic work above, we found that BCL2 had the strongest pattern, with three sgRNAs in four replicate screens indicating that the effect of its knockout acts synergistically with FLT3 inhibition. We found some indication (though the pattern is not as strong) that the effect of knocking out MCL1 or BCL-X_L_ may also enhance the anti-tumor effect of FLT3 inhibition, though not to the same degree as with BCL2. Interestingly, we were also able to examine what happens when BAX or BAK is knocked out in the presence of FLT3 inhibition and found a pattern of a slight increase in sgRNA levels; this in effect acts like a genetic surrogate for sequestration of these proteins by BCL2 family members and supports the idea that decreasing their efficacy enhances the pro-survival aspect of AML blasts.

Because leukemia-initiating cells have been shown to aberrantly overexpress BCL2 and be preferentially killed by BCL2 inhibition, even when in a quiescent state [17, 18], we sought to characterize the effects of midostaurin plus venetoclax on blast re-plating potential using methylcellulose colony-forming unit (CFU) assays of primary patient cells. Samples were stratified by the presence or absence of *FLT3*-ITD as evidenced by capillary gel electrophoresis. Venetoclax alone strongly reduced the ability of primary AML cells to form colonies compared to control dimethyl sulfoxide, (DMSO; *p* < 0.001 for *FLT3*-ITD and *p* = 0.006 for *FLT3*-WT) (Fig. 2c, d), and we did not find a statistically significant difference between treatment with venetoclax and treatment with venetoclax plus midostaurin on the aggregate data. Although the dominant effect on colony-forming ability was from venetoclax, we sought to determine how many patient samples appeared to have any reduction of colony-forming units with the addition of midostaurin to venetoclax. Therefore, we normalized all of the combination treatment data to venetoclax treatment and found that seven of nine *FLT3*-ITD samples had a very small percent reduction in colony-forming units with the addition of midostaurin, suggesting that there could be a small benefit for some patients (Additional file 1: Fig. S1C). *FLT3*-WT patient samples did not exhibit any significant alterations with the addition of midostaurin to venetoclax over venetoclax alone. Re-plating potential was also decreased in all drugging conditions for all but one of the *FLT3*-ITD samples. For patients that grew colonies upon secondary plating, the combination of midostaurin and venetoclax outperformed single-agent effects in three of six *FLT3*-ITD samples, displayed approximately the same effect in two of six *FLT3*-ITD samples, and showed a slightly decreased effect in one of six *FLT3-*ITD samples (Additional file 1: Fig. S1D). While midostaurin had a very limited effect on colony-forming ability, gilteritinib hindered colony-forming ability similar to venetoclax in *FLT3*-ITD patients (Fig. 2c). Thus, the combination of gilteritinib and venetoclax did not appear to further reduce colonies compared to monotherapy conditions. Interestingly, colony reduction by gilteritinib was also seen in *FLT3*-WT patient samples (Fig. 2d). A few patients responded less robustly to venetoclax with no shared co-occurring mutations found to explain their differential response (Additional file 1: Fig. S2).

Following completion of elegant mechanistic studies, Ma et al. concluded with a xenograft mouse study (*n* = 5 per group) of gilteritinib and venetoclax in female immunocompromised triple transgenic NSGS non-obese diabetic/SCID gamma (NOD.Cg-Prkdc^scid^ Il2rg^tm1Wjl^ 219 Tg(CMV220 IL3, CSF2, KITLG)1Eav/MloySzJ) mice engrafted with MV4-11 cells. We sought to further these studies for clinical translatability with two separate AML mouse models, including a xenotransplantation model and an adoptive transfer of spontaneous murine AML.

First, MOLM-13 xenograft studies using cells tagged with luciferase enabled us to perform several new investigations, including visualization with IVIS imaging, survival analysis, and histopathologic examination. Given the different indications of gilteritinib and midostaurin (single agent in relapsed/refractory patients versus frontline treatment in combination with standard chemotherapy, respectively), we also expanded our studies to include the in vivo combination of midostaurin and venetoclax [8, 19]. Briefly explained, MOLM-13 luciferase cells were engrafted in NOD/SCID IL2rγ (NSG) mice and divided into four treatment arms: (1) vehicle, (2) midostaurin, (3) venetoclax, and (4) midostaurin/venetoclax combination (Fig. 3a). The estimated median survival time for mice given the midostaurin/venetoclax combination was 63 days, compared to 23 days for mice given venetoclax only (*p* = 0.004, log-rank test) or 41 days for mice given midostaurin only (*p* = 0.004, log-rank test) (Fig. 3b). IVIS imaging (Fig. 3c) and histopathologic analysis of the bone marrow, lung, and liver (Additional file 1: Fig. S3) showed disease burden consistent with observed survival. A similar synergy was also observed using gilteritinib in combination with venetoclax. NOD-*Prkdc*^*em26Cd52*^*Il2rg*^*em26Cd22*^/NjuCrl (NCG) mice were transplanted with MOLM-13 luciferase cells and divided into vehicle, venetoclax, gilteritinib, and combination arms (Fig. 3d). Mice receiving the combination of gilteritinib and venetoclax had an improved survival compared to gilteritinib alone (log-rank *p* < 0.001, Fig. 3e, f).

**Fig. 3:**
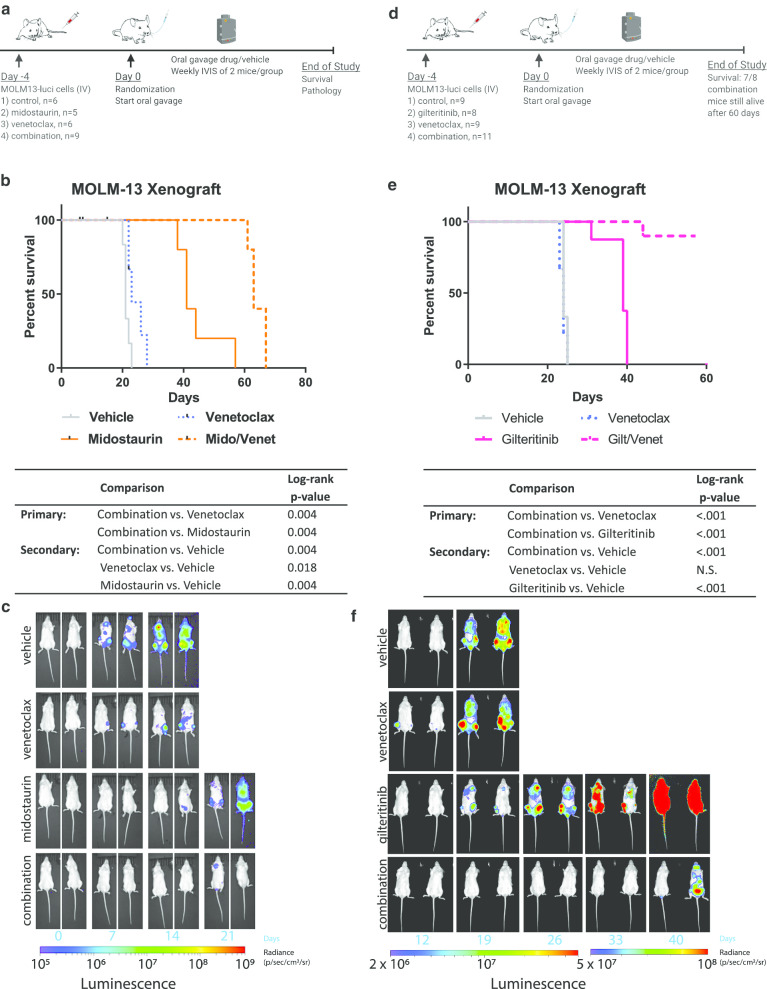
In vivo validation of the combination of venetoclax and FLT3 inhibitors **a** NSG mice were engrafted with MOLM-13 cells expressing luciferase and treated with vehicle, 50 mg/kg midostaurin, 75 mg/kg venetoclax, or both midostaurin and venetoclax. **b** Kaplan–Meier analysis of the mouse survival. The log-rank test was used to compare survival between groups of interest. Comparisons of single agent vs. combo were considered primary; all comparisons versus vehicle were considered secondary. *p* values were adjusted for multiple comparisons using Holm’s method for the primary and secondary comparisons separately. **c** IVIS imaging shows changes in luciferase signal over 3 weeks. **d** NCG mice were engrafted with MOLM-13 cells expressing luciferase and treated with vehicle, 30 mg/kg gilteritinib, 75 mg/kg venetoclax, or both gilteritinib and venetoclax. **e** Kaplan–Meier analysis of the mouse survival. Statistics were performed as described for the midostaurin/venetoclax experiment. **f** IVIS imaging shows changes in luciferase signal over 5 weeks

Next, we tested similar drug combinations using an adoptive transfer model, engrafting cells from two donor mice that spontaneously develop AML due to *FLT3*-ITD and TET2 knockout [20] into NCG mouse recipients (*n* = 8–17 mice/group). Such spontaneous models offer an alternative important way to model therapeutic response independent of cell lines conditioned to grow in vitro. Mice were block randomized into six treatment arms upon engraftment: (1) vehicle, (2) midostaurin, (3) venetoclax, (4) midostaurin/venetoclax, (5) gilteritinib, and (6) gilteritinib/venetoclax (Fig. 4a).

**Fig. 4:**
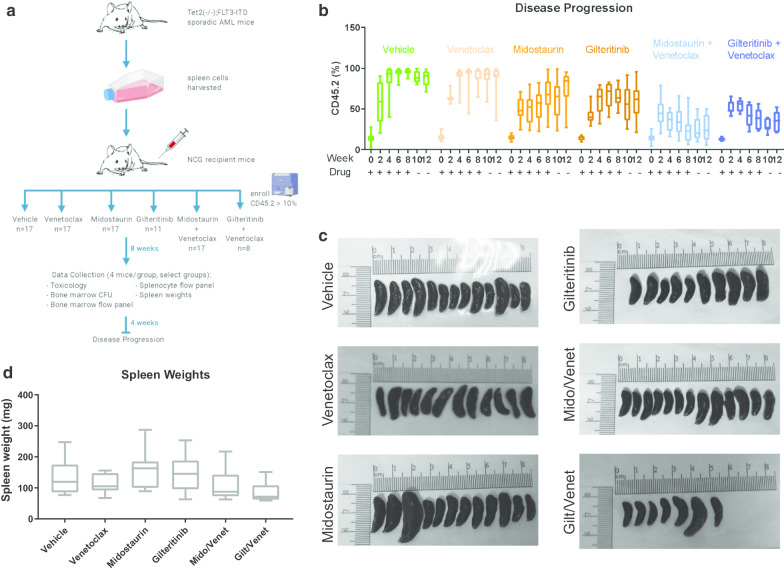
Adoptive transfer AML murine model from *Tet2*^−/−^*Flt3*^ITD^ mice treated with venetoclax and FLT3i. **a** Study design. **b** Disease progression was monitored by bimonthly flow cytometry of CD45.2 + (donor) cells. **c** Spleens were excised and measured at the end of the study. **d** Spleens were weighed at the end of the study

Because NCG mice exclusively express CD45.1, whereas donor mice express only CD45.2, changes in disease burden were analyzed bimonthly to monitor percent CD45.2 in the peripheral blood of recipients. Over an 8-week treatment course, both vehicle and venetoclax-only mice reached nearly 100% CD45.2 in peripheral blood. Treatment with either midostaurin or gilteritinib slowed disease progression leading to an average disease burden of about 70% CD45.2. After 2–4 weeks of treatment, average percent CD45.2 began to decrease in both combination groups and by 8 weeks approached 20% in midostaurin/venetoclax and 40% in gilteritinib/venetoclax (Fig. 4b). All drug treatments were stopped at 8 weeks, and mice were followed for an additional 4 weeks, during which period midostaurin monotherapy-treated mice experienced a modest increase in disease burden, while gilteritinib and both combination groups mostly maintained stable disease (Fig. 4b). For the duration of the experiment, we did not see a significant change in white blood cell counts (Additional file 1: Fig. S4A) or treatment-associated weight loss (Additional file 1: Fig. S4B) in any groups. Mice were killed and examined for changes in splenomegaly as reported in this model [20]. One midostaurin monotherapy-treated mouse and one gilteritinib/venetoclax combination therapy-treated mouse had very large spleens (Fig. 4c) with other spleens demonstrating a trend toward smaller average size in combination therapy mice compared to other arms of the study (Fig. 4d).

After 8 weeks of treatment, four mice from midostaurin monotherapy, venetoclax monotherapy, midostaurin/venetoclax, and vehicle groups were killed for interim analysis. We found no evidence of drug-associated toxicologic lesions in any tissues examined for midostaurin or midostaurin/venetoclax therapy (Additional file 1: Fig. S4C). Additionally, we found that average spleen weight decreased in the midostaurin/venetoclax combination group compared to each monotherapy or vehicle (Additional file 1: Fig. S4D, S4E). Colony-forming assays of the recovered bone marrow demonstrated lower survival of cells from the midostaurin/venetoclax combination therapy mice compared to those recovered from each monotherapy or vehicle mice (Additional file 1: Fig. S4F). Overall, the results of the spontaneous murine model support further clinical development of gilteritinib/venetoclax and midostaurin/venetoclax combination therapies.

Using multiple, orthogonal approaches, we validated the synergistic relationship between the effects of BCL2 and FLT3 inhibition that was predicted by our previous work [11] and expanded preclinical work by Ma et al. [12] using genetic validation and clinical-grade inhibitors that could be more readily translated to the clinic as combination strategies. We showed that depletion of BCL2 via both gene silencing and chemical inhibition in addition to FLT3 inhibition increased cell death in *FLT3*-ITD cell lines and primary AML patient samples beyond that of each perturbation alone, an effect which was not observed to the same extent in the case of *FLT3*-WT. Furthermore, we demonstrated that combination therapy with venetoclax plus midostaurin or gilteritinib can improve survival in xenograft and adoptive transfer murine AML models, examining disease progression, recovered bone marrow cells, and survival. The work presented here supports further clinical development of the combinations of midostaurin and venetoclax as well as gilteritinib and venetoclax and specifically highlights the reproducibility of the synergistic effect across multiple disease-replicating contexts (Additional file 2).

## Supplementary information

**Additional file 1.** Additional figures.

**Additional file 2.** Methods.

## Data Availability

CRISPR screen data referenced during this study are included in the published article [11], and data are available in the Gene Expression Omnibus via accession number GSE143314.

## Abbreviations

AML: Acute myeloid leukemia
BAK: BCL2 antagonist/killer
BAX: BCL2-associated X protein
BCL2: B-cell lymphoma 2
BCL-X_L_: B-cell lymphoma-extra large
CFU: Colony-forming unit
CRISPR: Clustered regularly interspaced short palindromic repeats
DMSO: Dimethyl sulfoxide
FLT3: FMS-like tyrosine kinase 3
ITD: Internal tandem duplication
KD: Knockdown
MCL1: Myeloid cell leukemia 1
NCG: NOD-*Prkdc*^*em26Cd52*^*Il2rg*^*em26Cd22*^/NjuCrl
NSG: NOD/SCID gamma
NSGS: NOD.Cg-Prkdc^scid^ Il2rg^tm1Wjl^ 219 Tg(CMV220 IL3, CSF2,KITLG)1Eav/MloySzJ
PDGFR: Platelet-derived growth factor receptors
sgRNA: Single guide ribonucleic acid
STAT5: Signal transducer and activator of transcription 5
TKD: Tyrosine kinase domain
XPO1: Exportin 1
WT: Wild type
