# Supplementary material for: Synergistic effect of BCL2 and FLT3 co-inhibition in acute myeloid leukemia
Source: J Hematol Oncol. 2020 Oct 19;13:139. doi: 10.1186/s13045-020-00973-4 (PMC7574303; doi:10.1186/s13045-020-00973-4)
Supplement: Supplementary file 1 — Additional file 1. Additional figures. [file 13045_2020_973_MOESM1_ESM.pptx]

## Slide 1
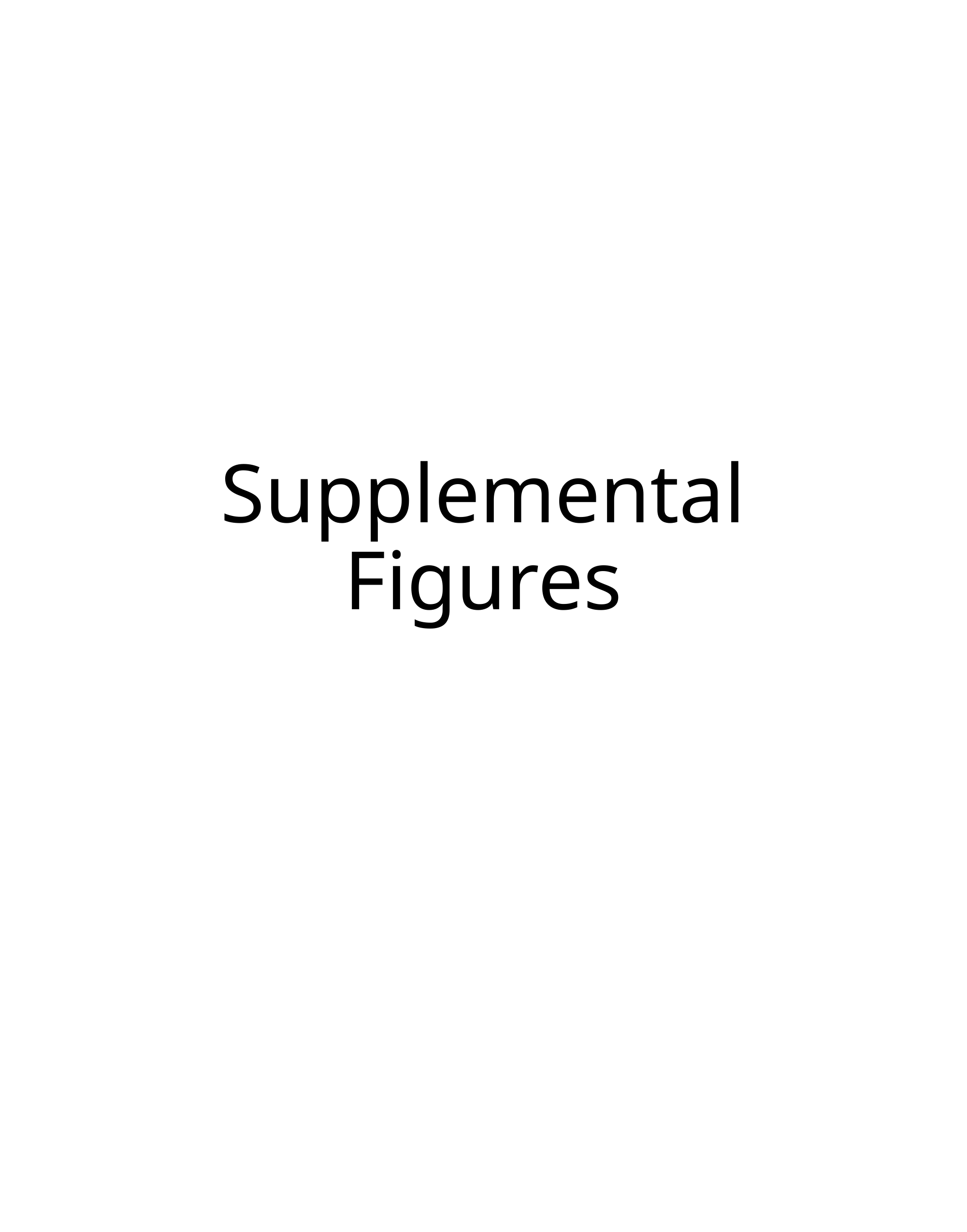

# Supplemental Figures

## Slide 2
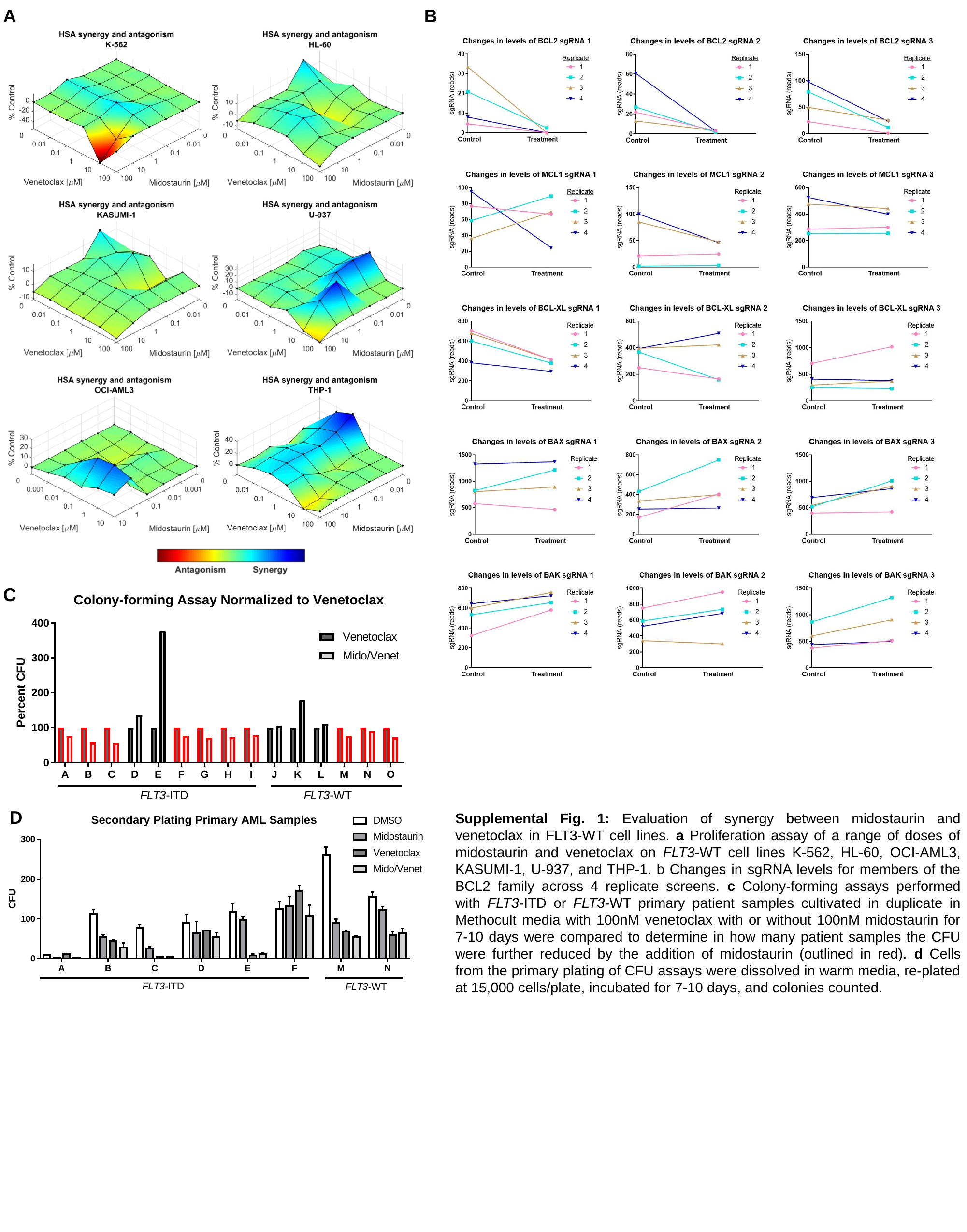

A
B
C
D
Supplemental Fig. 1: Evaluation of synergy between midostaurin and venetoclax in FLT3-WT cell lines. a Proliferation assay of a range of doses of midostaurin and venetoclax on FLT3-WT cell lines K-562, HL-60, OCI-AML3, KASUMI-1, U-937, and THP-1. b Changes in sgRNA levels for members of the BCL2 family across 4 replicate screens. c Colony-forming assays performed with FLT3-ITD or FLT3-WT primary patient samples cultivated in duplicate in Methocult media with 100nM venetoclax with or without 100nM midostaurin for 7-10 days were compared to determine in how many patient samples the CFU were further reduced by the addition of midostaurin (outlined in red). d Cells from the primary plating of CFU assays were dissolved in warm media, re-plated at 15,000 cells/plate, incubated for 7-10 days, and colonies counted.

## Slide 3
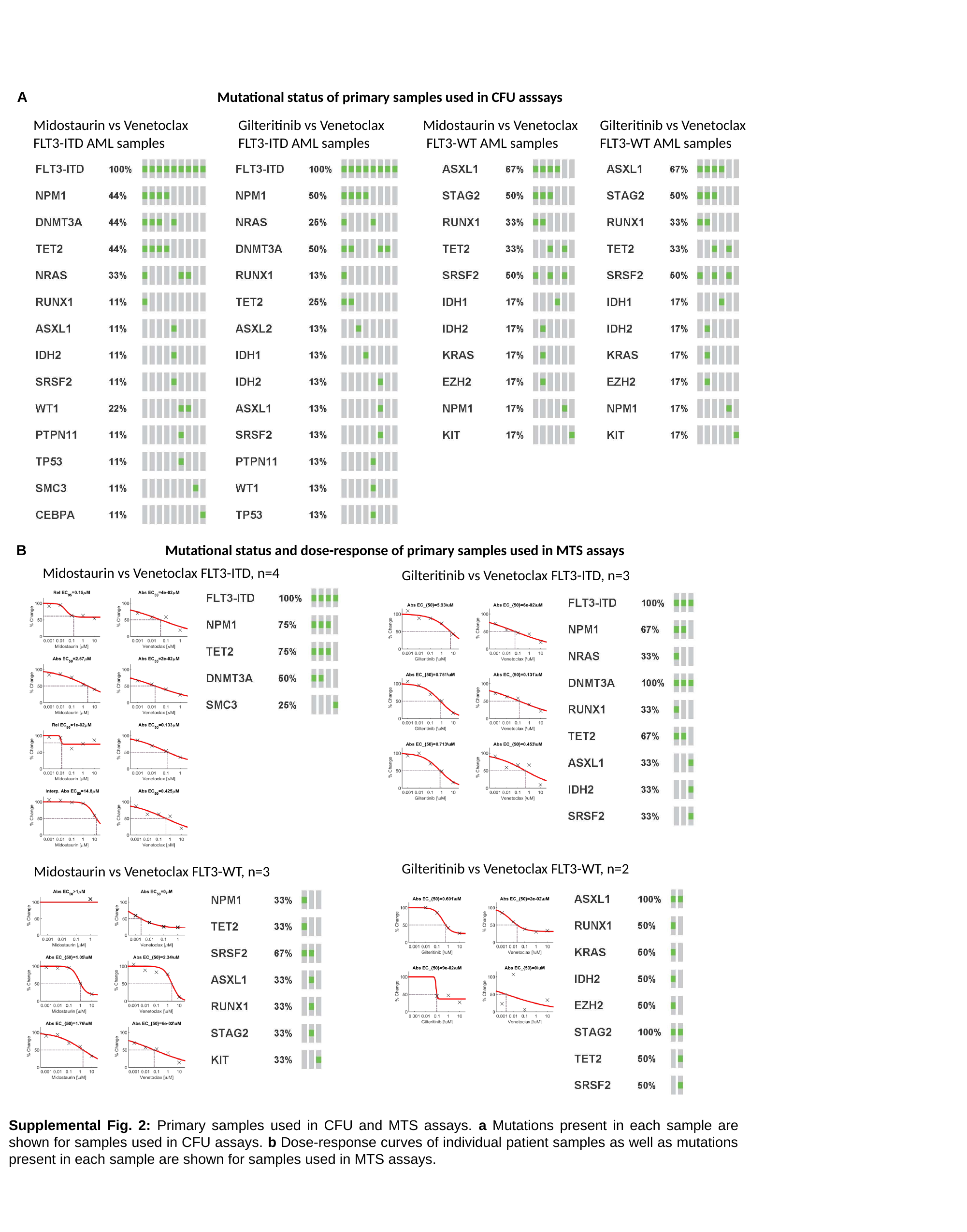

A
Mutational status of primary samples used in CFU asssays
Midostaurin vs Venetoclax
FLT3-ITD AML samples
Gilteritinib vs Venetoclax
FLT3-ITD AML samples
Midostaurin vs Venetoclax
 FLT3-WT AML samples
Gilteritinib vs Venetoclax
FLT3-WT AML samples
Mutational status and dose-response of primary samples used in MTS assays
B
Midostaurin vs Venetoclax FLT3-ITD, n=4
Gilteritinib vs Venetoclax FLT3-ITD, n=3
Gilteritinib vs Venetoclax FLT3-WT, n=2
Midostaurin vs Venetoclax FLT3-WT, n=3
Supplemental Fig. 2: Primary samples used in CFU and MTS assays. a Mutations present in each sample are shown for samples used in CFU assays. b Dose-response curves of individual patient samples as well as mutations present in each sample are shown for samples used in MTS assays.

## Slide 4
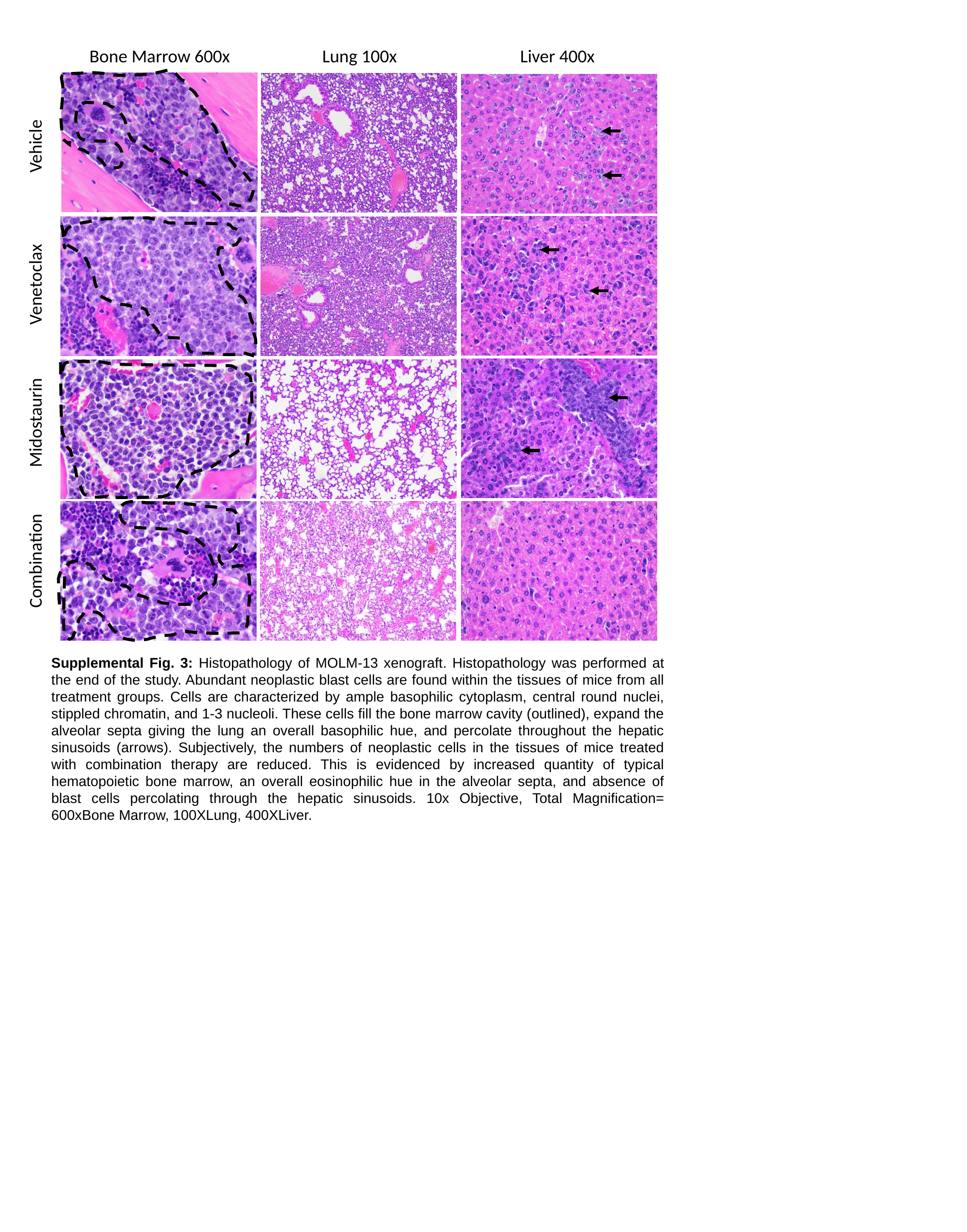

Bone Marrow 600x
Lung 100x
Liver 400x
Vehicle
Venetoclax
Midostaurin
Combination
Supplemental Fig. 3: Histopathology of MOLM-13 xenograft. Histopathology was performed at the end of the study. Abundant neoplastic blast cells are found within the tissues of mice from all treatment groups. Cells are characterized by ample basophilic cytoplasm, central round nuclei, stippled chromatin, and 1-3 nucleoli. These cells fill the bone marrow cavity (outlined), expand the alveolar septa giving the lung an overall basophilic hue, and percolate throughout the hepatic sinusoids (arrows). Subjectively, the numbers of neoplastic cells in the tissues of mice treated with combination therapy are reduced. This is evidenced by increased quantity of typical hematopoietic bone marrow, an overall eosinophilic hue in the alveolar septa, and absence of blast cells percolating through the hepatic sinusoids. 10x Objective, Total Magnification= 600xBone Marrow, 100XLung, 400XLiver.

## Slide 5
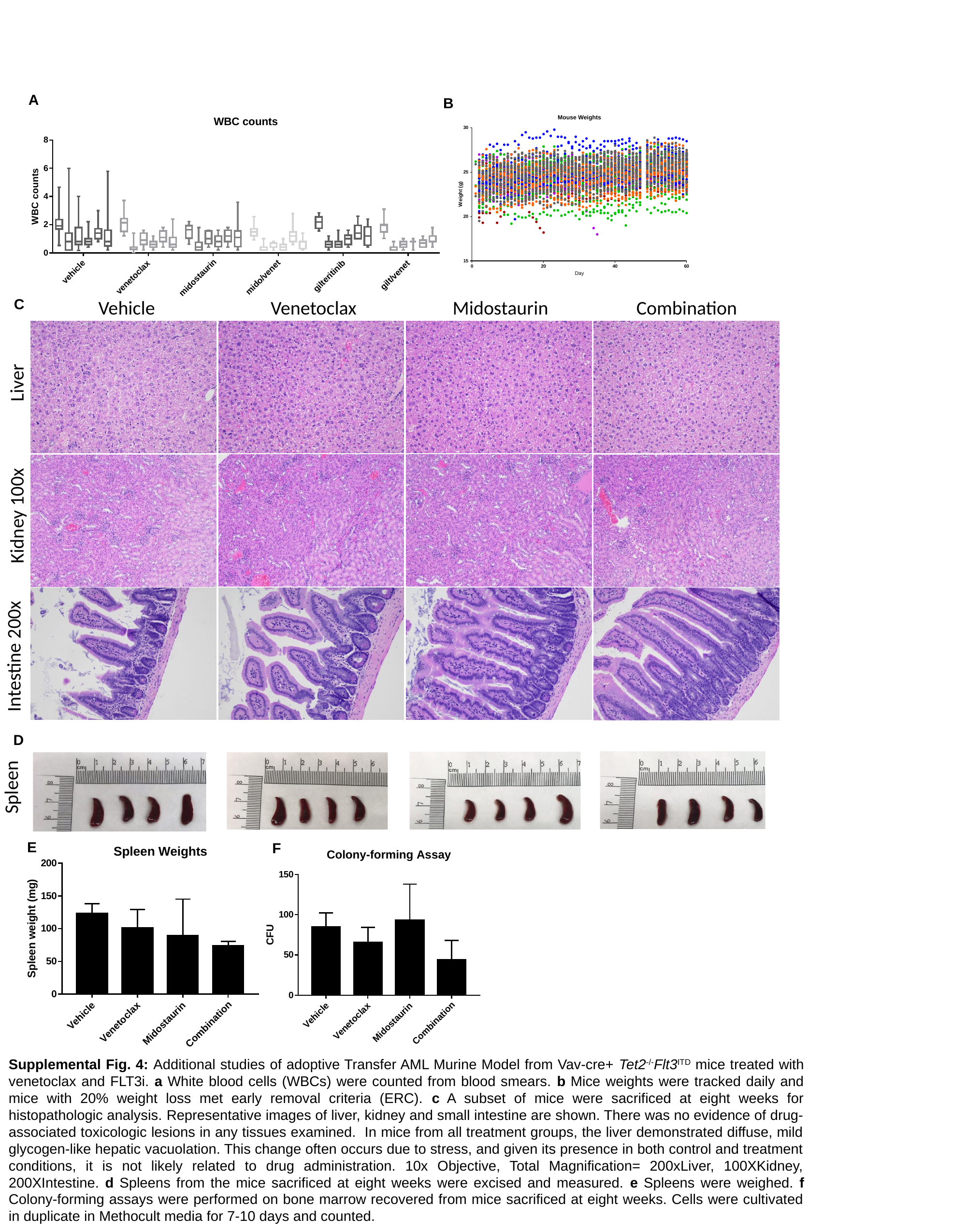

A
B
C
Vehicle
Venetoclax
Midostaurin
Combination
Liver 200x
Kidney 100x
Intestine 200x
D
Spleen
E
F
Supplemental Fig. 4: Additional studies of adoptive Transfer AML Murine Model from Vav-cre+ Tet2-/-Flt3ITD mice treated with venetoclax and FLT3i. a White blood cells (WBCs) were counted from blood smears. b Mice weights were tracked daily and mice with 20% weight loss met early removal criteria (ERC). c A subset of mice were sacrificed at eight weeks for histopathologic analysis. Representative images of liver, kidney and small intestine are shown. There was no evidence of drug-associated toxicologic lesions in any tissues examined. In mice from all treatment groups, the liver demonstrated diffuse, mild glycogen-like hepatic vacuolation. This change often occurs due to stress, and given its presence in both control and treatment conditions, it is not likely related to drug administration. 10x Objective, Total Magnification= 200xLiver, 100XKidney, 200XIntestine. d Spleens from the mice sacrificed at eight weeks were excised and measured. e Spleens were weighed. f Colony-forming assays were performed on bone marrow recovered from mice sacrificed at eight weeks. Cells were cultivated in duplicate in Methocult media for 7-10 days and counted.
