# Supplementary material for: Synergistic effect of BCL2 and FLT3 co-inhibition in acute myeloid leukemia
Source: J Hematol Oncol. 2020 Oct 19;13:139. doi: 10.1186/s13045-020-00973-4 (PMC7574303; doi:10.1186/s13045-020-00973-4)
Supplement: Supplementary file 2 — Additional file 2. Methods. [file 13045_2020_973_MOESM2_ESM.docx]

**Supplement**

**Methods**

***Cell culture***

MOLM-13, MV4-11, OCI-AML3, U-937, K-562, HL-60, HS5-GFP, and THP-1 cells were cultured in filtered RPMI 1640 (Gibco) supplemented with 10% Fetal Bovine Serum (FBS, VWR). HEK293FT cells were cultured in DMEM (Gibco) with 10% FBS. Kasumi-1 were cultured in RPMI with 20% FBS. All cell lines were supplemented with 1% penicillin/streptomycin/glutamine. HS5-GFP cells were a kind gift from Dr. William Dalton (H. Lee Moffitt Cancer Center). MOLM-13-luciferase cells were a kind gift from Dr. Ramiro Garzon (Ohio State University). MOLM-13, MV4-11, and OCI-AML3 cell lines were purchased from Deutsche Sammlung von Mikroorganismen und Zellkuturen (Braunschweig, Germany) in February 2015. HEK293FT cells were purchased from Life Technologies (R70007). All other cell lines were purchased from American Type Culture Collection (Manassas, VA) in January 2015. All cell lines routinely tested negative for mycoplasma contamination (Universal Mycoplasma Detection Kit, ATCC 30-1012K), validated by Microsatellite genotyping (short tandem repeat analysis by the Ohio State University Genomic Services Core), and used between passage five and twenty.

***CRISPR ribonucleoprotein knockout***

Ribonucleoprotein (RNP) complexes were formed as directed by manufacturer using Alt-R CRIPSR-Cas9 cRNA, tracrRNA, and Cas9 (Integrated DNA Technologies). MOLM-13 cells were electroporated with RNP and Alt-R Cas9 electroporation enhancer in Solution SF (Lonza V4XC-2012) on a Lonza 4D-Nucleofector System with pulse code EH100, using Alt-R CRISPR –Cas9 sgRNAs against BCL2: AGGGCGATGTTGTCCACCAG, TGGCGCACGCTGGGAGAACA, GGGGCCGTACAGTTCCACAA.

***Immunoblot Analysis***

Protein molecules were size-separated by gel electrophoresis from whole-cell lysates, then transferred onto nitrocellulose membrane as previously described. (1) Antibodies included: anti- BCL2 clone 124 (Cell Signaling Technology 15071S) and anti-B-actin (Cell Signaling Technology 4967S). Western blots were quantified using ImageJ band densitometry analysis.

***Proliferation Assays***

MTS assays were performed to determine cell proliferation as previously described. (1)

***Primary AML sample proliferation and colony-forming unit (CFU) assays***

Cyropreserved primary cells with or without *FLT3*-ITD from bone marrow, peripheral blood, or post-apheresis of AML patients with diverse co-occurring mutations (**Supplemental Fig. 2**) were obtained from the Ohio State University Comprehensive Cancer Center Leukemia Tissue Bank or gifted from Greg Behbehani. Proliferation assays on primary cells were conducted by plating 1×10^5^ cells in 96-well plates with one of the following support conditions: (1) hypoxic conditions with plates coated with rat tail Collagen Type 1 (Life Technologies) and media supplemented with a cytokine cocktail of hGM-CSF, hSCF, hFLT3-L, and hIL-3 (PeproTech) at 10ng/mL each, or (2) normoxic co-culture with HS5-GFP stromal cells. Drugs were added in increasing concentrations and incubated 96-hours. CellTiter 96 (Promega) was added and formazan dye quantified by absorbance (490nm) after 5 hours. For stromal co-culture, leukemic cells were transferred to a new plate before reading absorbance. For CFU assay, live cells were counted via trypan blue exclusion and plated at optimal density in two 35mm^2^ dishes in MethoCult H04035 Optimum without EPO (Stemcell) plus 0.1uM midostaurin, 0.05uM gilteritinib, 0.1uM venetoclax, combinations of drugs, or DMSO control. Colonies were counted after 7-14 days, re-plated at 15,000 cells/dish, and counted again after 7-14 days. Co-occurring mutations were determined by a next-generation sequencing targeted capture panel and visualized with Oncoprint.(2,3)

***Animal Studies***

All animal experiments were carried out under protocols approved by the Ohio State University Institutional Animal Care and Use Committee. For cell line xenograft, 10^4^ MOLM-13 luciferase-tagged cells were injected via tail vein of male or female NOD-*scid* IL2Rgamma^null^, NOD-*scid*IL2Rg^null^ (NSG) mice from the Jackson Laboratory or NOD-*Prkdc^em26Cd52^Il2rg^em26Cd22^*/NjuCrl (NCG) mice from the Charles River Laboratory. On day three post-engraftment, mice were randomized to treatment arms. For the FLT3^ITD^-TET2^-/-^ adoptive transfer model, mice were gifted by Ross Levine[^9^](https://www.zotero.org/google-docs/?kwoOeV) and 10^6^ splenocytes from these mice were injected into the tail vein of NCG mice from the Charles River Laboratory. Leukemia onset was defined as ≥10% CD45.2 cells in peripheral blood by flow cytometry. At leukemia onset, engrafted mice were assigned to treatment groups by block randomization, with covariate 1 as the donor mouse and covariate 2 as the percent of CD45.2. Overall survival was the primary end point for the majority of the mice, with 16 mice allotted for microscopic pathology analysis after eight weeks of drug-treatment.

Mice received the following drugs and doses: 75 mg/kg venetoclax (ChemieTek CT-A199) daily gavage in 1 part EtOH, 3 parts PEG400, and 6 parts Phosal 50PG; 50 mg/kg midostaurin (Selleck Chemicals S8064 for xenograft study; MedChemExpress HY-10230 for adoptive transfer model) daily gavage in 6% drug w/w gelucire(R)44/14 (Gattefosse, France), which was aliquoted and mixed with drug weekly; 30mg/kg gilteritinib (MedChemExpress HY-12432) daily gavage in 6% drug w/w gelucire(R)44/14 (Gattefosse, France), which was aliquoted and mixed with drug weekly. Aliquots of gelucire were stored at 4^o^C and warmed each day to 44^o^C in a heat block, then diluted with sterile water to a concentration of 12 mg/mL.

Mice were weighed daily prior to treatment to determine appropriate dose and received a maximum volume of 200 uL via oral gavage. Vehicle mice in the xenograft study received only EtOH/PEG400/Phosal PG. Mice in the adoptive transfer model all received both vehicles (double gavage of EtOH/PEG400/Phosal PG and gelucire with or without drug, depending on the group). Mice were monitored by animal technicians who were blinded to treatment groups and determined when mice met Early Removal Criteria (20% weight loss, lethargy, palor, labored breathing, hunching, poor body condition). At 10% weight loss, mice were given a two-day drug holiday; in the adoptive transfer study, mice with 15% weight loss were also isolated.

***Statistical Analysis***

For BCL2 KD experiments, mixed effects models were applied to the data to account for dependencies among observations from the same biological replicate; models included the interaction between parent/KD and drug. Synergy ratio calculations were performed as described previously. (4) For CFU assays, negative binomial models were fit to the count data and differences between conditions assessed. For mouse survival experiments, the primary endpoint was overall survival (OS). Median survival time in each group was estimated using Kaplan-Meier methods and differences in OS between groups assessed using the log-rank test. Highest Single Agent analyses were performed using Combenefit software. (5) All other analyses were performed using SAS/STAT software, version 9.4 of the SAS System for Windows (SAS Institute, Inc., Cary, NC).

**References**

1. Ozer HG, El-Gamal D, Powell B, Hing ZA, Blachly JS, Harrington B, et al. BRD4 Profiling Identifies Critical Chronic Lymphocytic Leukemia Oncogenic Circuits and Reveals Sensitivity to PLX51107, a Novel Structurally Distinct BET Inhibitor. Cancer Discov. 2018 Apr 1;8(4):458–77.

2. Cerami E, Gao J, Dogrusoz U, Gross BE, Sumer SO, Aksoy BA, et al. The cBio cancer genomics portal: an open platform for exploring multidimensional cancer genomics data. Cancer Discov. 2012 May;2(5):401–4.

3. Gao J, Aksoy BA, Dogrusoz U, Dresdner G, Gross B, Sumer SO, et al. Integrative analysis of complex cancer genomics and clinical profiles using the cBioPortal. Sci Signal. 2013 Apr 2;6(269):pl1.

4. Brinton LT, Sher S, Williams K, Canfield D, Orwick S, Wasmuth R, et al. Cotargeting of XPO1 Enhances the Antileukemic Activity of Midostaurin and Gilteritinib in Acute Myeloid Leukemia. Cancers. 2020 Jun;12(6):1574.

5. Veroli D, Y G, Fornari C, Wang D, Mollard S, Bramhall JL, et al. Combenefit: an interactive platform for the analysis and visualization of drug combinations. Bioinformatics. 2016 Sep 15;32(18):2866–8.
